# Prospective pilot study on the relationship between seminal HIV-1 shedding and genital schistosomiasis in men receiving antiretroviral therapy along Lake Malawi

**DOI:** 10.1038/s41598-023-40756-8

**Published:** 2023-08-29

**Authors:** Sekeleghe A. Kayuni, Adam Abdullahi, Mohammad H. Alharbi, Peter Makaula, Fanuel Lampiao, Lazarus Juziwelo, E. James LaCourse, Johnstone J. Kumwenda, Peter Derek Christian Leutscher, Anna Maria Geretti, J. Russell Stothard

**Affiliations:** 1 Department of Tropical Disease Biology, Liverpool School of Tropical Medicine, Liverpool, L3 5QA UK; 2 MASM Medi Clinics Limited, Medical Aid Society of Malawi (MASM), Lilongwe, Malawi; 3 Malawi Liverpool Wellcome (MLW) Clinical Research Programme, Kamuzu University of Health Sciences (KUHeS), Queen Elizabeth Central Hospital Campus, Chipatala Avenue, Blantyre, Malawi; 4 Department of Pathology, School of Medicine and Oral Health, Kamuzu University of Health Sciences, Mahatma Gandhi Road, Blantyre, Malawi; 5 Department of Global Health and Population, Harvard T.H Chan School of Public Health, Boston, MA USA; 6 Cambridge Institute of Therapeutic Immunology and Infectious Diseases, University of Cambridge, Cambridge, UK; 7 Ministry of Health, Buraydah, 52367 Saudi Arabia; 8 Research for Health, Environment and Development (RHED), Mangochi, Malawi; 9 Physiology Unit, Department of Biomedical Sciences, School of Life Sciences and Allied Health Professions, Kamuzu University of Health Sciences, Mahatma Gandhi Road, Blantyre, Malawi; 10 National Schistosomiasis and Soil-Transmitted Helminths Control Programme, Community Health Sciences Unit, Ministry of Health, Lilongwe, Malawi; 11 Department of Internal Medicine, School of Medicine and Oral Health, Kamuzu University of Health Sciences, Mahatma Gandhi Road, Blantyre, Malawi; 12 Centre for Clinical Research, North Denmark Regional Hospital and Department of Clinical Medicine, Aalborg University, Aalborg, Region Nordjylland Denmark; 13 Department of Infectious Diseases, Fondazione PTV, University of Rome Tor Vergata, Rome, Italy; 14 School of Immunology and Microbial Sciences, King’s College London, London, UK

**Keywords:** Diseases, Medical research

## Abstract

Male genital schistosomiasis (MGS) is hypothesized to increase seminal shedding of HIV-1. This prospective pilot study assessed seminal HIV-1 RNA shedding in men on long-term ART with and without a diagnosis of MGS. Study visits occurred at 0, 1, 3, 6 and 12 months. MGS was diagnosed by egg positivity on semen microscopy or PCR of seminal sediment. After optimization of the HIV-RNA assay, we examined 72 paired plasma and semen samples collected from 31 men (15 with and 16 without MGS) over 12 months. HIV-1 RNA was detected in 7/72 (9.7%) seminal samples and 25/72 (34.7%) plasma samples. When comparing sample pairs, 5/72 (6.9%) showed HIV-1 RNA detection only in the seminal sample. Overall, 3/31 (9.7%) participants, all with MGS, had detectable HIV-1 RNA in semen while plasma HIV-1 RNA was undetectable (< 22 copies/mL), with seminal levels ranging up to 400 copies/mL. Two participants showing HIV-1 RNA in seminal fluid from the MGS-negative group also had concomitant HIV-1 RNA detection in plasma. The findings suggest that MGS can be associated with low-level HIV-1 RNA shedding despite virologically suppressive ART. Further studies are warranted to confirm these observations and assess its implications.

## Introduction

Three-quarters of the global burden of HIV-1 infection resides within sub-Saharan Africa^1^ which also bears a disproportionately high burden of neglected tropical diseases. Here, schistosomiasis, a parasitic infection caused by water-borne blood flukes^2^, afflicts some 180 million people whereas an estimated 6 million are living with HIV-1^3,4^. Although levels of co-infection of schistosomiasis and HIV are not accurately reported, an increased risk of HIV-1 acquisition may be assumed in those with underlying schistosomiasis^5^.

Indeed, HIV-1 prevalence is often raised in fishing communities where schistosomiasis is prevalent^6,7^. In endemic areas, there is a higher prevalence of HIV-1 infection in women with female genital schistosomiasis (FGS), and schistosomiasis has been shown to increase the risk of HIV-1 acquisition by a factor of three^8–10^. This association is due to genital mucosal breach, neovascularisation, and increased density of HIV receptive CD4 + cells in women with FGS. Genital schistosomiasis has also been associated with increased seminal levels of interleukin (IL)-4, IL-6, IL-10, and tumour necrosis factor-alpha in men with seminal egg excretion^11^. However, there are limited data on the relationship between male genital schistosomiasis (MGS) and HIV-1 infection among men. A study in Zimbabwe examined four antiretroviral therapy (ART)-naïve men living with HIV-1 and demonstrated a decline in seminal HIV-1 RNA levels (by a median of 0.6 log_10_ copies/mL) 10 weeks after treatment with praziquantel (PZQ), the only available medicine against schistosomiasis^12^.

ART is highly effective in suppressing HIV-1 replication in both plasma and genital tract, reducing the risk of HIV-1 transmission through both heterosexual and homosexual intercourse. Prospective studies of HIV-discordant couples where the partner living with HIV was established on virologically suppressive ART (defined as plasma HIV-1 < 200 copies/mL) showed no HIV-1 transmission despite condomless sex^13–16^. Nonetheless, detection of HIV-1 RNA in seminal fluid has been previously reported in men receiving virologically suppressive ART^17,18^. One explanation is that HIV-1 RNA suppression occurs more slowly in seminal fluid than in plasma and thus seminal shedding may be relatively common in the early phase after ART initiation^19^. Furthermore, it has been hypothesised that like sexually transmitted and other genital infections, MGS may promote genital shedding of HIV-1 RNA despite effective ART due to chronic egg-induced inflammation of the genital tract^20^.

In Malawi, within a population of 19.9 million, 990 000 (5%) were estimated to be living with HIV-1 in 2021^21^. Whilst epidemiological data is generally heterogenous across sub-Saharan Africa, in 2015, one study in Malawi reported schistosomiasis prevalence of 47.4% in communities around its ﻿lakeshores and other environmental water bodies^22^. The aim of this pilot study was to assess the extent of any putative relationship between MGS and seminal HIV-1 RNA shedding among men living with HIV-1 and receiving long-term ART in Malawi.

## Methods

### Population and sampling

**Figure 1:**
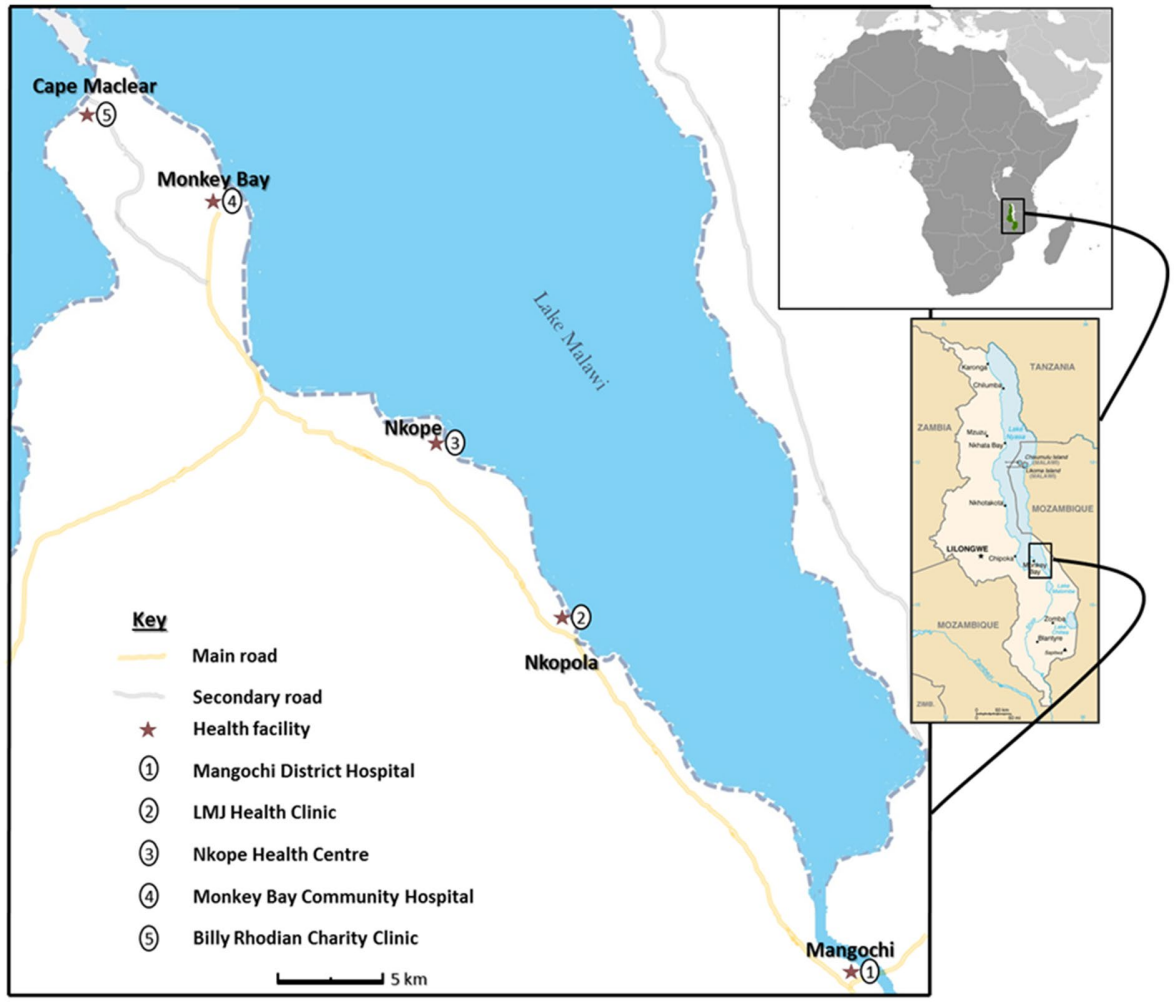
Schematic map of study area showing health facilities along the shores of Lake Malawi (https://www.cia.gov/library/publications/the-world-factbook/attachments/locator-maps/MI-locator-map.gif and https://www.cia.gov/library/publications/the-world-factbook/attachments/maps/MI-map.gif)^24^.

Study participants were heterosexual men ≥ 18 years of age living with HIV-1 who attended HIV outpatient services along Lake Malawi between October 2017 and December 2018 (Fig. 1). Mid-morning urine, semen and whole blood in EDTA were to be collected at each planned study visit (0, 1, 3, 6 and 12 months). The sampling methodology was reported previously^23^ and is described in Supplementary Information. Seminal samples were collected in a clear plastic bag following two days of abstinence from sexual activity. In total, 74 participants enrolled, of which 23 did not submit blood or urine samples for schistosomiasis testing. Of the remaining 51, 33 tested negative for schistosomiasis, of whom 16 provided blood, urine, and semen. This cohort of 16 was deemed the ‘MGS-negative group'. Of the 18 participants that tested positive for schistosomiasis, 15 provided blood, urine, and semen. This cohort of 15 was deemed the ‘MGS-positive group'. The disposition of the whole study population is shown in Supplementary Fig. 1. Given the high prevalence of schistosomiasis in the region^22^, participants received PZQ at all study visits regardless of a diagnosis of MGS.

### Laboratory procedures

Samples were processed within 3 hours of collection. Whole blood samples were centrifuged at 3000 *xg* for 5 minutes to separate plasma for storage at −80°C. Seminal samples were allowed to liquefy at ambient temperature and examined under microscopy for the presence of *Schistosoma* eggs, followed by centrifugation at 3000 *xg* for 5 minutes to separate supernatant seminal fluid and sediments. Seminal fluid was stored at −80°C. Seminal sediment samples were re-dissolved in saline and 2–3 drops were placed on a glass slide for microscopy to detect *Schistosoma* eggs. Leftover seminal sediments were preserved in ethanol and shipped to the Elisabeth-TweeSteden Ziekenhuis (ETZ) Hospital in Tilburg, the Netherlands, for *Schistosoma* DNA detection by in-house real-time PCR as previously described^23^. The parasitological procedure was previously detailed^23^ and is described in Supplementary Information. Urine samples were tested for *Schistosoma* circulating cathodic antigen (CCA) using the point-of care parasite CCA test (POC-CCA) (Rapid Medical Diagnostics, South Africa), followed by urine filtration and microscopy for *Schistosoma* eggs. Cryopreserved seminal fluid and plasma samples were shipped on dry ice to the United Kingdom for HIV-1 RNA testing.

#### Schistosomiasis diagnosis and definition of MGS

*Schistosoma* positivity was defined by visual evidence of eggs by microscopy of filtrated urine, semen, or seminal sediment; PCR detection in seminal sediment, urine or stool; or positive POC-CCA test in urine. Male genital schistosomiasis (MGS) was defined as *Schistosoma* positivity in semen.

#### HIV-1 RNA testing

HIV-1 RNA was detected in plasma and seminal fluid using the Cepheid Xpert assays^25^. The assays perform qualitative (HIV-1 Qual assay) and quantitative (HIV-1 Viral Load assay) detection of HIV-1 RNA in plasma^26^. With 1 mL input, the HIV-1 Qual assay reports qualitative HIV-1 RNA detection with a lower limit of detection (LLOD) of 278 copies/mL; the manufacturer describes 25% detection rate at 60 copies/mL^27,28^. With 1mL input, the HIV-1 Viral Load assay has a lower limit of quantification (LLOQ) of 40 copies/mL and a LLOD of 22 copies/mL^26^. To detect HIV-1 RNA in seminal fluid, the assays were first validated using seminal fluid samples collected from two HIV negative donors and spiked with known amounts of HIV-1 RNA, as detailed in Supplementary Information. The assay LLOD for seminal fluid ranged from 55 to 220 copies/mL according to the dilution applied. HIV-1 RNA results were reported as either a quantified level or as qualitative targeted detected.

### Statistical analyses

Baseline characteristics were described as categorical and continuous variables and compared using Mann–Whitney–Wilcoxon test (continuous variables) or Fisher’s exact test (categorical variables). Analyses was performed using IBM SPSS Statistics (version 27) and GraphPad Prism version 8.0.0.

### Ethical considerations

Ethical approval was provided by the National Health Sciences Research Committee (NHSRC) of Malawi (Approval No.: 1805) and the Liverpool School of Tropical Medicine Research Ethics Committee (Approval No.: 17-018). Participants provided written informed consent to be recruited and participate in the study in accordance with the Declaration of Helsinki. All research methods were performed in accordance with relevant guidelines and regulations. All participants reserved the right to opt-out at any stage of the study.

## Results

### Study population and sampling

A total of 31 participants established on ART provided at least one paired semen and blood sample over the 12 months of the study. Their baseline characteristics are summarised in Table 1. At study entry, participants had received ART for a median of 7.5 years (IQR 1.9–13.1). Most were receiving coformulated tenofovir disoproxil fumarate/lamivudine/efavirenz (TDF/3TC/EFV). A total of 72 paired samples were collected, with a median of 2 paired samples per participant (range 1–5), including 30 samples at study entry (baseline), and 10, 12, 9 and 11 samples at 1, 3, 6 and 12 months. Eleven participants donated only one set of paired samples. Overall, based on the date of the last paired samples, the median duration of follow-up was 10.2 months (IQR 4.0–14.2). All except 6 participants (one with MGS and 5 without) had received PZQ in the 12 months prior to recruitment. None showed symptoms suggestive of sexually transmitted infections (STIs) and no samples were collected to investigate asymptomatic STIs.

**Table 1:** Characteristics of study participants at study entry.

Characteristic	Total	MGS status		*p*-value
		Positive	Negative	
Total number (%)	31	15	16	
Age, median years (IQR)	46 (39, 52)	42.0 (36, 46)	51 (43, 59)	0.78
Praziquantel in the previous 12 months, n (%)	25 (80.6)	14 (93.3)	11 (68.7)	0.17
Years since HIV diagnosis, median (IQR)	7.4 (1.4, 11.7)	7.5 (11.0, 14.4)	7.8 (6.3, 11.3)	0.50
ART regimen, n (%)				
TDF/3TC/EFV	25	13	12	0.53
TDF/3TC + NVP	2	1	1	–
Not known	4	1	3	–
ART duration, median years (IQR)	7.5 (1.9, 13.1)	5.5 (1.9, 12.0)	7.5 (5.9, 12.8)	0.44
Plasma HIV-1 RNA, n (%)				
Not detectable	21 (67.7)	9 (60.0)	12 (75.0)	0.69
Detectable	9 (29.0)	5 (33.3)	4 (25.0)	
Not available	1 (3.2)	1 (6.7)	0 (0)	
Seminal HIV-1 RNA, n (%)				
Not detectable	27 (87.1)	12 (80.0)	15 (93.7)	0.59
Detectable	3 (9.7)	2 (13.3)	1 (6.3)	
Not available	1 (3.2)	1 (6.7)	0 (0)	

MGS, Male Genital Schistosomiasis; IQR, Interquartile range; ART, Antiretroviral therapy; TDF, Tenofovir disoproxil fumarate; 3TC, Lamivudine; EFV, Efavirenz; NVP, Nevirapine.

### MGS status

Details of parasitology testing are summarised in Table 2. At baseline, 8 participants tested MGS positive by semen microscopy, 4 tested positive only by real-time PCR of seminal sediment, and 20 tested negative by all tests. Among the 20 participants with a negative baseline test, 3 had a positive test during follow-up (1 by real-time PCR only; 1 by PCR and POC-CCA test and 1 by urine filtration, PCR and POC-CCA test) yielding a total of 15 participants who were classed as MGS positive (A01–A15), whereas 16 were classed as MGS negative (A16–A31). There were no significant differences when comparing the baseline characteristics of the two groups (Table 1). During the study period, 42 paired samples were provided by the MGS positive participants while 30 samples were provided by MGS negative participants.

**Table 2:**
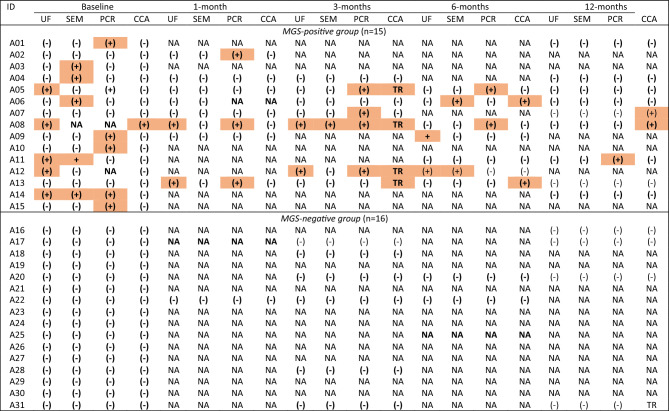
Overview of parasitology testing.^a^

^a^Positive results are shadowed; bold text indicates timepoint with corresponding paired HIV-1 RNA testing. UF, Urine filtration and microscopy; SEM, Semen microscopy; CCA, Circulating cathodic antigen; NA, Not available; (−), Negative; (+), Positive; TR, Trace; MGS, Male genital schistosomiasis.

### HIV-1 RNA testing

#### Plasma

At baseline, 5/30 (16.7%) participants showed quantifiable plasma HIV-1 RNA ($\ge$ 40 copies/mL) including 3/14 (21.4%) in the MGS-positive group (A09, A11, A15) and 2/16 (12.5%) in the MGS-negative group (A21, A30) (Table 3). HIV-1 RNA levels ranged from 41 to 56,000 copies/mL During follow-up, 1/5 participants (A09) achieved suppression < 40 copies/mL at 6 months, while continuing to show detectable HIV-1 RNA below the assay LLOQ of 40 copies/mL (estimated 22–39 copies/mL); 1/5 (A11) showed persistent viraemia between 816 and 277 copies/mL at 6 and 12 months; and 3/5 had no follow-up samples collected. A further 4 participants showed detectable plasma HIV-1 RNA below the assay LLOQ of 40 copies/mL (estimated 22–39 copies/mL) at baseline, including 2/14 (14.3%) in the MGS-positive group (A07, A13) and 2/16 (12.5%) in the MGS-negative group (A16, A23). Among 21 participants with undetectable HIV-1 RNA at baseline, two in the MGS-negative group showed plasma viral load rebound > 40 copies/mL at 1 month (A17; 64 copies/mL) and 3 months (A20; 107,000 copies/mL) respectively, followed by resuppression < 40 copies/mL) but detectable HIV-1 RNA at 12 months (Table 3).

**Table 3:**
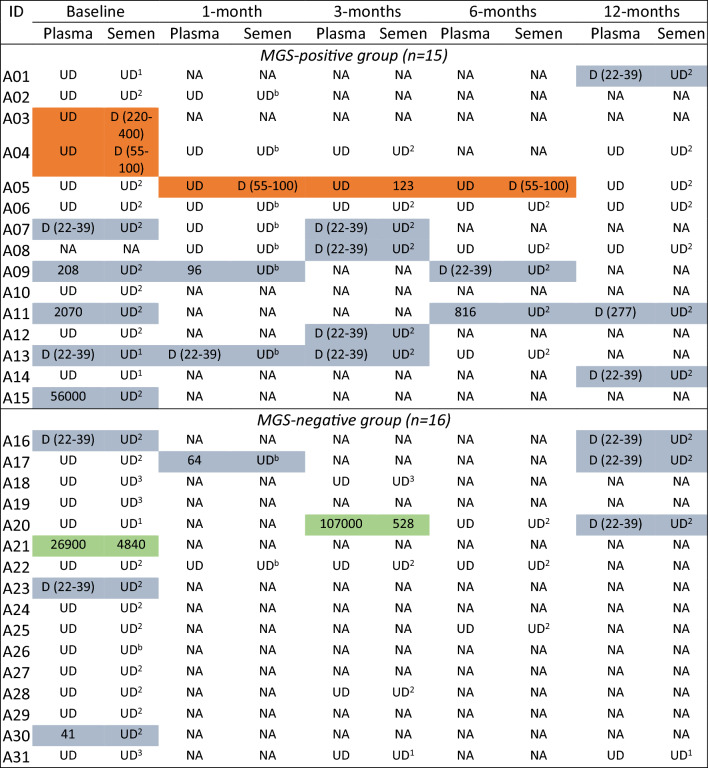
HIV-1 RNA testing results (in copies/mL) in plasma and seminal fluid by MGS status.^a^

^a^With plasma, HIV-1 RNA results were either quantifiable ($\ge$ 40 copies/ml), detectable qualitatively (D, estimated level 22–39 copies/ml), or undetectable (UD, < 22 copies/ml); with seminal fluid, the lower limit of detection varied according to the sample dilution and is specified by sample as follows (the estimated HIV-1 RNA levels are given in parenthesis): UD^1a^ = HIV-1 RNA undetectable (< 110 copies/ml); UD^2b^ = HIV-1 RNA undetectable (< 55 copies/ml); UD^3c^ = HIV-1 RNA undetectable (< 220 copies/ml); D = HIV-1 RNA detected (viral load in copies/ml).

Shadowing legend

Orange color: HIV-1 RNA detectable in semen and undetectable in plasma.

Blue color: HIV-1 RNA detectable in plasma and undetectable in semen.

Green color: HIV-1 RNA detectable in plasma and semen.

#### Seminal fluid

Among participants with baseline samples, 3/30 (10.0%) showed detectable HIV-1 RNA in seminal fluid (Table 3). In the MGS-positive group, 2/14 (14.3%) participants (A03, A04) showed detectable HIV-1 RNA (estimated levels between 55 and 400 copies/mL) in seminal fluid, while plasma HIV-1 RNA was undetectable (< 22 copies/mL). One of the two had no follow-up samples. The other showed undetectable HIV-1 RNA in both plasma and seminal fluid at months 1, 3 and 12. In the MGS-negative group, 1/16 (6.3%) participants (A21) showed seminal HIV-1 RNA levels of 4840 copies/mL, but in this case plasma HIV-1 RNA levels were also high at 26,900 copies/mL. A total of 20 participants had at least one follow-up sample (Table 3). Among these, 2 participants (A05, A20) showed newly detectable HIV-1 RNA in seminal fluid. One participant (A05) in the MGS-positive group had persistent HIV-1 RNA in seminal fluid at months 1, 3 and 6, with levels ranging up to 123 copies/mL, while plasma HIV-1 RNA remained persistently undetectable (< 22 copies/mL). The second participant (A20) was in the MGS-negative group. At 3 months, HIV-1 RNA levels were 528 copies/mL in seminal fluid while plasma HIV-1 RNA had rebounded from undetectable to 107,000 copies/mL. Overall, 7/72 (9.7%) seminal fluid samples had detectable or quantifiable HIV-1 RNA, comprising 3 samples at baseline (2 in the MGS-positive group) and 1, 2 and 1 samples at 1, 3 and 6 months, respectively (3 in the MGS-positive group).

#### Patterns of HIV-1 RNA detection by MGS status

When comparing the 72 paired plasma and seminal samples, HIV-1 RNA detection (with or without quantification) was concordant in 44/72 (61.1%) samples (Table 4). The remaining 28/72 (38.9%) sample pairs were discordant; most had HIV-1 RNA detected in plasma only. In the MGS-negative group, 2/16 participants had detectable HIV-1 RNA in 2 seminal samples, and in both cases the paired plasma showed high HIV-1 RNA levels. In contrast, in the MGS-positive group, 3/15 participants had detectable HIV-1 RNA in 5 seminal samples, and in all cases the paired plasma showed undetectable HIV-1 RNA. The characteristics of the 3 participants are detailed in the Table 5. They had been established on ART long-term, ranging between 8 and 12 years at study entry. Overall, 4 of the 5 seminal samples with detectable HIV-1 RNA had concomitant *Schistosoma* test positivity.

**Table 4:** Summary of the patterns of HIV-1 RNA detection and quantification in 72 paired plasma and seminal fluid samples.

Plasma^a^	Seminal fluid^b^		
	Targeted not detected^a^ (n)	Target detected (n)	Quantified (n)
Target not detected^b^	42	4	1
Target detected	15	0	0
Quantified	8	0	2

^a^The lower limit of quantification (LLOD) was 40 copies/mL; the lower limit of detection (LLOD) was 22 copies/mL. ^b^The LLOQ and LLOD depended on the available sample volume and therefore the dilution required to make up 1 mL input.

**Table 5:** Characteristics of the three participants with MGS showing detectable HIV-1 RNA in seminal fluid while plasma HIV-1 RNA was undetectable.

ID	Age (years)	ART regimen	ART duration (years)	Study timepoint (months)	Schistosomiasis status^a^	HIV-1 RNA			
						Plasma^b^	Copies/ml	Seminal fluid^c^	Copies/ml
A03	49	TDF/3TC/EFV	8.0	Baseline	+ ve SEM	Undetectable	< 22	Detectable	220–400
A04	47	TDF/3TC/EFV	11.9	Baseline	+ ve SEM	Undetectable	< 22	Detectable	55–100
			12.1	1	−ve	Undetectable	< 22	Undetectable	< 55
			12.3	3	−ve	Undetectable	< 22	Undetectable	< 55
			13.1	12	−ve	Undetectable	< 22	Undetectable	< 55
A05	43	TDF/3TC/EFV	11.9	Baseline	+ve UF; + ve PCR (Ct = 36.1)	Undetectable	< 22	Undetectable	< 55
			12.1	1	Negative	Undetectable	< 22	Detectable	55–100
			12.3	3	+ ve PCR: (Ct = 22.5)	Undetectable	< 22	Quantified	123
			12.5	6	+ ve PCR: (Ct = 23.4)	Undetectable	< 22	Detectable	55–100
			13.1	12	−ve	Undetectable	< 22	Undetectable	< 55

^a^Schistosomiasis status: + ve SEM, Positive semen microscopy; + ve UF, Positive urine filtration; + ve PCR, Positive real-time polymerase chain reaction; −ve, Negative for all the three tests, namely UF, SEM, and PCR. ^b^The lower limit of quantification (LLOQ) was 40 copies/mL; the lower limit of detection (LLOD) was 22 copies/mL. ^c^The LLOQ and LLOD depended on the available sample volume and therefore the dilution required to make up 1 mL input.

## Discussion

Across sub-Saharan Africa, evidence of the impact of MGS on HIV-1 shedding in semen is limited despite the significant epidemiological overlap between HIV-1 infection and endemic schistosomiasis^29^. We sought to assess prospectively over 12 months, the rates and kinetics of seminal HIV-1 RNA shedding in heterosexual men living with HIV-1 and established on first-line NNRTI-based ART in Malawi, stratifying the data according to a diagnosis of MGS. In our pilot study, we observed low-level HIV-1 RNA shedding in seminal fluid of men with MGS despite fully suppressed plasma HIV-1 RNA, whereas in those without an MGS diagnosis seminal HIV-1 RNA shedding was always concomitant with high-level viremia. Our numbers are limited. In total, we observed HIV-1 RNA detection in 5 seminal samples from 3 of 15 participants in the MGS group. Larger studies are needed to confirm these interesting observations.

Urogenital schistosomiasis is thought to be similar to STIs such as HSV-2 in facilitating HIV-1 transmission; this is due to presence of mucosal lesions and local recruitment of HIV-susceptible cells through egg-induced inflammation^3^. Collectively, this is expected to increase the risk of HIV acquisition and propagation by increasing viral replication. Whilst data reporting the impact of urogenital schistosomiasis on HIV-1 shedding in semen are scarce, one study showed a decrease in seminal HIV-1 RNA levels following treatment with PZQ during a follow-up period of 10 weeks^12^.

Virologically effective ART reduces the risk of transmitting HIV sexually via both heterosexual and homosexual routes^14,15,30^. One question remains as to the relevance of the reported delay in HIV-1 RNA suppression in seminal fluid relative to plasma in the early phase of ART. In our study, participants entered the analysis while already established on ART with a median ART duration of 7.5 years (IQR 1.9–13.0). In this population established on long-term ART, 5/31 (16.1%) participants had at least one episode of seminal HIV-1 RNA detection over 12 months of follow-up. Two of the participants were negative for *Schistosoma* and had concomitant high HIV-1 RNA detection in plasma. Among participants positive for *Schistosoma,* 3 were established on ART with TDF/3TC/EFV for 8–12 years and had fully suppressed, undetectable HIV-1 RNA in plasma, yet they showed detectable HIV-1 RNA in a total of 5 seminal samples. It is important to highlight that the levels of seminal HIV-1 RNA in these 5 samples were low and never exceeded an estimated 400 copies/mL, despite 4 of the 5 samples coinciding with a positive *Schistosoma* test. The implications in terms of risk of HIV-1 transmission are uncertain.

As a pilot study, we acknowledge several limitations. The study population was small and participants did not provide samples at all scheduled time points. We had small seminal sample volumes which constrained our ability to use a high input volume in the HIV-1 RNA tests. Due to lack of samples, we did not attempt to perform HIV drug resistance testing when HIV-1 RNA was detected and we did not investigate the concomitant occurrence of clinically unrecognised STIs.

## Conclusion

Taken together, our study found that 3/15 (20%) men with HIV-1 and MGS who were established on NNRTI-based first-line ART for at least 8 years had detectable HIV-1 RNA in 5 seminal samples while plasma HIV-1 RNA was fully suppressed. Seminal HIV-1 RNA shedding coincided with *Schistosoma* detection in 4 of the 5 samples. However, HIV-1 RNA was detected in seminal fluid at low levels, raising doubts about significance in terms of risk of transmission, particularly if shedding is sporadic or intermittent. We recommend that future studies should aim to (1) evaluate the definitive role of MGS in enhancing HIV-1 RNA shedding in semen during ART; (2) investigate the infectiousness and drug resistance profile of seminal HIV-1 RNA; and (3) determine how the introduction of dolutegravir-based ART across Africa may impact on the findings especially as reports have started to observe treatment failure across populations^31,32^.

### Supplementary Information

Supplementary Information.

## Data Availability

All data generated or analysed during this study are included in this manuscript and its supplementary information files. Any additional information can be reasonably requested from the corresponding author.
