# Supplementary material for: Prospective pilot study on the relationship between seminal HIV-1 shedding and genital schistosomiasis in men receiving antiretroviral therapy along Lake Malawi
Source: Sci Rep. 2023 Aug 29;13:14154. doi: 10.1038/s41598-023-40756-8 (PMC10465494; doi:10.1038/s41598-023-40756-8)
Supplement: Supplementary file 1 — Supplementary Information. [file 41598_2023_40756_MOESM1_ESM.docx]

**Supplementary Information**

Description of the Study methodology

Supplementary Figure 1. Study flow and patient disposition

Supplementary Table 1. Summary of the validation of the Cepheid Xpert HIV assay for HIV-1 RNA detection

Supplementary Table 2. Characteristics of the fifteen patients with plasma HIV-1 RNA detection while seminal HIV-1 RNA was fully suppressed (<22 copies/mL)

Supplementary Table 3. Characteristics of the fourteen patients with concordant HIV-1 RNA detection in both seminal fluid and plasma.

**Description of the Study methodology**

***Study area, population and sampling***

The research study was conducted among fishermen living in fishing communities (villages) identified and selected along the south shoreline of Lake Malawi in Mangochi district from October 2017 to December 2018. Mangochi is the largest district in the southern region of Malawi, covering 6,729 km2 of land with at least 1.1 million people (NSO, 2018).

The district has a tropical continental climate with a longer dry season of cold weather from May to August and hot weather from September to November, and a relatively shorter wet season from December to April (NSO, 2011). Most fishermen in the area live in specific fishing villages, closer to the lake to carry out their routine fishing related activities.

This was a longitudinal cohort study, comprising of baseline surveys of MGS among fishermen and follow-up studies after praziquantel treatment, conducted in villages and nearby health centres. Fishermen aged ≥ 18 years willing to provide written informed consent were eligible to participate in the study. Using the estimated 20% prevalence of *S. haematobium* in adults from previous studies and assuming 10% having MGS, a minimum sample size of 275 fishermen (adjusted for assumed 10% loss to follow-up), was planned to be randomly selected for the study to measure the current prevalence of MGS and subsequent follow-up studies (Kirkwood and Sterne, 2006; CDC, 2014).

***Data collection and analysis***

The following are the data collection methods and analyses that were used in the study:

***Individual questionnaires***

After briefing about the study and obtaining written informed consent, fishermen were recruited in their communities and interviewed with individual questionnaires, collecting information on demographic, health, hygiene, sanitation and socio-economic characteristics. This information assessed their knowledge, perceptions, attitudes and practices on MGS and HIV. The questionnaires were developed from standardised questions administered elsewhere in a similar study (Ukwandu and Nmorsi, 2004).

The questionnaires were piloted on the first 10 participants to assess the reliability of the questions. After the questionnaire interviews, the participants were invited to the nearby health facility to submit urine, semen and for ultrasonography examination.

***Parasitological analyses***

The recruited study participants were provided with a clean sample container at the health facility to submit urine, between 10am and 2pm for filtration to examine for schistosome eggs to confirm urogenital schistosomiasis (UGS). Semen was submitted in a clear, transparent, self-sealing plastic bag after abstaining from coitus for two days to examine for male genital schistosomiasis (MGS), defined in the study as the presence of schistosome eggs in semen.

***Urine analysis - filtration***

Urine was analysed immediately for macrohematuria by visual inspection using a urine colour card, and then for microhematuria, leukocytes and proteinuria using reagent strips (Siemens multistix 10G) and scores were recorded in the following categories: negative, trace, +, ++ and +++.

Point-of-care circulating cathodic antigen (POC-CCA) test was conducted on the urine to assess for possible intestinal infection by *S. mansoni*, following manufacturer’s instructions (Rapid Medical Diagnostics, South Africa; batch no. 171103130) and as described previously (van Dam et al., 2004). Urine was measured and recorded accordingly, before conducting filtration following approved standard guidelines (WHO, 1991; Cheesbrough, 2009).

The entire volume of urine was filtered through a disinfected filter containing a clean polycarbonate membrane with 20 μm pores to trap as many S. haematobium eggs in the sample. The membrane was removed, placed on a standard glass slide and examined under the microscope.

Iodine was added to visualise the eggs distinctly. The number of eggs was calculated by first, dividing the total eggs observed by the total volume filtered and then multiplying by 10. The resultant egg count was recorded per 10 ml of urine. Highest infection intensity for UGS was defined as egg count of ≥ 50 eggs per 10 ml urine as widely described (Cheesbrough, 2009).

***Seminal microscopic analysis***

After submission, the bag with semen was placed under room temperature on a clean bench surface to allow the semen to liquefy. Thereafter, the semen was pushed gently to one corner of the clear plastic bag. Then the bag was heat-sealed to evenly concentrate the semen for easy visualization during microscopy. Direct examination of the semen bag was conducted under a microscope to check for schistosome eggs and the presence of leukocytes (WHO, 2010), thereafter the results were recorded as per ml of ejaculate.

Afterwards, the semen was measured and centrifuged at 3000 *xg* for 5 minutes to collect the seminal plasma. The sediment was re-dissolved in 0.5 ml normal saline for wet mount inspection using 2-3 drops and placed on a slide with a coverslip for microscopy, followed by recording of the results. Thereafter, 0.5 ml of ethanol was added to the remaining sediment for preservation and stored together with the seminal plasma at -80°C in preparation for shipment to the United Kingdom for real-time polymerase chain reaction of *Schistosoma* genus DNA and HIV viral load for those participants on ART.

***HIV-1 RNA testing***

HIV-1 RNA in collected plasma and seminal fluid was performed using the Cepheid Xpert assay (1). The Cepheid Xpert assay is optimised for qualitative (HIV-1 Qual assay) and quantitative (HIV-1 Viral Load assay) detection of HIV-1 in plasma (2). With 1 ml input, the HIV-1 Qual assay reports qualitative HIV-1 RNA detection with a lower limit of detection (LLOD) of 278 copies/ml; the manufacturer describes 25% detection rate at 60 copies/ml (3). With 1ml input, the HIV-1 Viral Load assay has a lower limit of quantification (LLOQ) of 40 copies/ml and a LLOD of 22 copies/ml (2). To detect HIV-1 RNA in seminal fluid, the Cepheid Xpert assay was first validated using seminal fluid samples collected from two HIV negative donors. Validation experiments were performed with the Xpert HIV-1 Qual assay using a two-step experimental approach. Samples were spiked with the third WHO reference standard for HIV-1 RNA (NIBSC 10/152) reconstituted with molecular grade water to a concentration of 185,000 IU/ml; this is equivalent to 107,558 copies/ml using a conversion factor of 1 HIV RNA copy/mL=1.72 IU/ml) (4). The final testing volume for testing was always 1ml. In the first set of experiments, samples were spiked to yield a final HIV-1 RNA concentration of 10000, 1000 and 100 copies/ml. This was achieved by starting with 100µl of seminal plasma, adding 93µl, 9.3µl and 0.93µl of the reference standard, and then adding water to a final 1ml volume. Due to the limited sample, each dilution point was performed as single test. In the second set of experiments, the concentration of HIV-1 RNA was kept at 291 copies/ml whereas the proportion of seminal fluid to diluent was varied. A total of 200, 300, 400, 500 and 900µl of sample were spiked with 2.7µl of reference standard, and then water was added to a final 1ml volume. This second set of experiments was performed with and without a spin step. For experiments including a spin step, samples were spiked, diluted and then centrifuged at 500rpm for 2 minutes prior to testing. Due to the limited sample, each dilution point was performed as single test. Cycle threshold (Ct) values, i.e., the threshold where fluorescence signal exceeded the background signal, were recorded and evaluated. Following validation, the detection of HIV-1 RNA in clinical samples was performed using the Xpert HIV-1 Viral Load assay. The first set of experiments showed no loss of detection in highly diluted samples and the second set of experiments showed no loss of HIV-1 RNA detection due to inhibition at seminal fluid input volume ≥400µl yielding an optimal detection dilution of 2.5 folds (Supplementary Table 1). Based on sample availability, between 100µl and 400µl of seminal plasma were diluted with water to a total 1ml volume, yielding a dilution factor between 10 and 2.5 folds. The assay LLOQ and LLOD were 400 copies/ml and 220 copies/ml respectively for 10-fold dilutions and 100 copies/ml and 55 copies/ml for 2.5-fold dilutions.

***UCP-LF CAA seminal analysis***

A trichloroacetic acid (TCA) extraction was performed on the seminal plasma following standard methods used for serum with an equal volume of 4% w/v TCA (Corstjens *et al.*, 2008). Small volume extraction (50 μl seminal plasma with 50 μL TCA) in microfuge tubes resulted in a clear supernatant after centrifugation (5 min, 13,000 rpm). The UCP-LF CAA analysis was performed according to standard methods with 20 μl of the clear supernatant, with a cut-off threshold of 10 pg/ml.

High volume extraction (0.5 ml seminal plasma with 0.5 ml TCA and a cut-off threshold of 1 pg/ml) required extended centrifugation time (30 min) before a clear supernatant was obtained; the resulting pellet was not rigid. Amicon 10 kDa centrifugal filtration devices (Merck Millipore) were used to concentrate 0.5 ml of the clear supernatant targeting concentration to 20 μl following standard methods used for serum undergoing centrifugation for 30 min at 13,000 rpm (Corstjens *et al.*, 2014).

***Schistosoma DNA real-time PCR analysis***

The ethanol preserved semen sediment was defrosted and centrifuged for 1 minute at 10,000 rpm. The ethanol layer was removed, and the pellet washed twice with 1 ml of phosphate buffered saline (PBS). The pellet was suspended in 0.4 ml of PBS containing 2% polyvinylpolypyrrolidone (PVPP) (Sigma, Steinheim, Germany). The suspension was heated for 10 min at 95°c and stored frozen overnight at -20°C.

DNA was extracted using the QIA symphony DSP virus / pathogen midi kit and pathogen complex 400 protocol of the QIA symphony Sample Processing (SP) system (Qiagen, Hilden, Germany). In each sample, a fixed amount of Phocine Herpes Virus 1 (PhHV-1) was added within the isolation lysis buffer, to serve as an internal control for the isolation procedure and to monitor inhibition of the real-time PCR. Schistosoma genus-specific real-time PCR was performed using primers and probes as described previously (Obeng et al., 2008; Kenguele et al., 2014), using threshold cycle (Ct-value) of 45.

***Ultrasonography examination***

Participants were briefed on the transabdominal and scrotal ultrasonography procedures to be conducted on them using a portable Chison Q5 ultrasound scanner with 3.5MHz probe supplied by Mount International United Services Ltd, Gloucester, United Kingdom. Participants were asked to present with a full bladder, before the procedure to increase the visualisation and validity of the images. The participant was positioned supine on the examination couch with the scanner set up on their right side. Whenever possible, room lightning was turned off to maximise screen visibility.

The scanning procedure investigated the appearance, size and abnormalities of the following key pelvic and genital organs: urinary bladder (shape, thickness, calcifications, masses, polyps), seminal vesicles (symmetry, thickness, nodules, echogenicity) and scrotum (tests, epididymis: nodules, masses, calcifications, hydroceles), according to evidence-based recommendations (Vilana *et al.*, 1997; WHO, 2000; Martino *et al.*, 2014). The observations made during the procedure and degree of visualisation were recorded accordingly.

All clips and images were stored on the device before transferring to the external hard drive for further analyses. A sample of 15% of the scan images were randomly selected and re-read by specialist radiologist for quality control, who conducted training of the study scanning personnel. All participants were notified of pathological findings that day, and further appropriate investigations and management were organised in accordance with standard clinical practice. Thereafter, praziquantel treatment at 40 mg/kg as a single dose was offered along with an invitation to follow-up studies after 1-, 3-, 6- and 12-months.

**Data analyses**

All the information collected during the study was screened and quality-controlled before entry into Microsoft Excel and SSPS computer packages. Screening for errors and cleaning were conducted, before commencing statistical analyses to present the results of the study.

**Inclusion criteria**:

HIV-1 positive, stable on ART

Men aged ≥ 18 years, including fishermen

Participants on ART invited to the MGS – HIV study

(n = **74**)

Participants recruited and had questionnaire interviews

(n = **74**)

Participants invited to submit urine and blood samples

(n = **51** [Schistosomiasis group: **18** + Non-schistosomiasis group: **33**])

Those who gave semen and received PZQ

(n = **31** [Schistosomiasis group: **15** + Non-schistosomiasis group: **16**])

**Health facility (group criteria)**:

Schistosomiasis group: either eggs in semen or urine or positive real-time PCR

Non-schistosomiasis group: no eggs in urine or semen, negative real-time PCR HIV-1 on ART only

**23** did not submit samples

**Health facility**:

All participants invited to submit semen

**20** did not submit semen

**Supplementary Figure 1. Study flow and patient disposition**

Supplementary Figure 2. Kinetics of HIV-1 RNA levels in plasma and semen following the initiation of antiretroviral therapy with tenofovir disoproxil fumarate (TDF), lamivudine (3TC) and efavirenz (EFV) in the participant with no schistosomiasis.

**Supplementary Table 1.** Summary of the validation of the Cepheid Xpert HIV assay for HIV-1 RNA detection

| Sample input (μl) | Diluent input (μl) | Test outcome | Ct values | |
| --- | --- | --- | --- | --- |
|  |  |  | Spin | No spin |
| 900 | 100 | Failed | N/A | |
| 500 | 500 | Failed | N/A | |
| 400 | 600 | Successful | 36.2 | 36.5 |
| 300 | 700 | Successful | 36.5 | 36.8 |
| 200 | 800 | Successful | 36.5 | 36.3 |

**Supplementary Table 2.** Characteristics of the fifteen patients with plasma HIV-1 RNA detection while seminal HIV-1 RNA was fully suppressed (<22 copies/mL)

| **ID** | **Age (years)** | **ART regimen** | **ART duration (years)** | **Timepoint** | **Follow-up time (months)** | **Schistosomiasis status** | **HIV-1 RNA** | | | |
| --- | --- | --- | --- | --- | --- | --- | --- | --- | --- | --- |
|  |  |  |  |  |  |  | **Plasma** | **Copies/ml** | **Seminal fluid** | **Copies/ml^a^** |
| A01 | 46 | TDF+3TC+EFV | 1.7 | T1 | 0 | +ve PCR | UD | <22 | UD | <110 |
|  |  |  | 2.8 | T2 | 13.0 | -ve | Detected | <40 | UD | <55 |
| A07 | 65 | TDF+3TC+EFV | 9.8 | T1 | 0 | -ve | Detected | <40 | UD | <55 |
|  |  |  | 9.9 | T2 | 1.8 | -ve | UD | <22 | UD | <55 |
|  |  |  | 10.1 | T3 | 3.7 | +ve PCR | Detected | <40 | UD | <55 |
|  |  |  | 10.3 | T4 | 6.7 | -ve | No sample | | No sample | |
|  |  |  | 10.9 | T5 | 13.0 | -ve | No sample | | No sample | |
| A08 | 42 | TDF+3TC+EFV | 0.7 | T1 | 0 | +ve UF | UD | <22 | No sample | |
|  |  |  | 0.9 | T2 | 1.4 | +ve PCR | UD | <22 | UD | <55 |
|  |  |  | 1.0 | T3 | 2.9 | +ve UF, +ve SEM, +ve PCR | Detected | <40 | UD | <55 |
|  |  |  | 1.2 | T4 | 6.0 | +ve PCR | UD | <22 | UD | <55 |
|  |  |  | 1.8 | T5 | 12.4 | -ve | UD | <22 | UD | <55 |
| A09 | 42 | TDF+3TC+EFV | 2.3 | T1 | 0 | +ve PCR | Quantified | 208 | UD | <55 |
|  |  |  | 2.5 | T2 | 1.9 | -ve | Quantified | 96 | UD | <55 |
|  |  |  | 2.9 | T3 | 6.8 | + UF | Detected | <40 | UD | <55 |
| A11 | 35 | TDF+3TC+EFV | 0.1 | T1 | 0 | +ve UF, +ve SEM | Quantified | 2070 | UD | <55 |
|  |  |  | 0.7 | T2 | 6.6 | -ve | Quantified | 816 | UD | <55 |
|  |  |  | 1.2 | T3 | 13.1 | -ve | Quantified | 277 | UD | <55 |
| A12 | 39 | TDF+3TC+EFV | 7.5 | T1 | 0 | +ve UF, +ve PCR | UD | <22 | UD | <55 |
|  |  |  | 7.8 | T2 | 2.9 | +ve UF, +ve SEM | Detected | <40 | UD | <55 |
| A13 | 36 | TDF+3TC+EFV | 0.3 | T1 | 0 | -ve | Detected | <40 | UD | <110 |
|  |  |  | 0.4 | T2 | 1.8 | +ve PCR | Detected | <40 | UD | <55 |
|  |  |  | 0.6 | T3 | 3.6 | -ve | Detected | <40 | UD | <55 |
|  |  |  | 0.8 | T4 | 6.4 | -ve | UD | <22 | UD | <55 |
|  |  |  | 1.7 | T5 | 12.9 | -ve | No sample | | No sample | |
| A14 | 30 | TDF/3TC/NVP | 0.6 | T1 | 0 | +ve UF, +ve SEM, +ve PCR | UD | <22 | UD | <110 |
|  |  |  | 1.7 | T2 | 12.9 | -ve | Detected | <40 | UD | <55 |
| A15 | 37 | TDF+3TC+EFV | N/A | T1 | 0 | +ve PCR | Detected | 56000 | UD | <55 |
| A16 | 43 | TDF+3TC+EFV | 1.8 | T1 | 0 | -ve | Detected | <40 | UD | <55 |
|  |  |  | 1.9 | T2 | 1.6 | -ve | No sample | | No sample | |
|  |  |  | 2.1 | T3 | 4.3 | -ve | No sample | | No sample | |
|  |  |  | 2.7 | T4 | 11.0 | -ve | Detected | <40 | UD | <55 |
| A17 | 34 | TDF/3TC/NVP | 6.2 | T1 | 0 | -ve | UD | <22 | UD | <55 |
|  |  |  | 6.4 | T2 | 2.7 | -ve | Quantified | 64 | UD | <55 |
|  |  |  | 6.9 | T3 | 9.4 | -ve | Detected | <40 | UD | <55 |
| A20 | 66 | TDF+3TC+EFV | 11.3 | T1 | 0 | -ve | UD | <22 | UD | <110 |
|  |  |  | 11.4 | T2 | 1.6 | -ve | Quantified | 107000 | Quantified | 528 |
|  |  |  | 11.6 | T3 | 4.3 | -ve | Detected | <40 | UD | <55 |
|  |  |  | 12.2 | T4 | 11.0 | -ve | Detected | <40 | UD | <55 |
| A23 | 48 | TDF+3TC+EFV | N/A | T1 | 0 | -ve | No sample | | No sample | |
|  |  |  |  | T2 | 1.6 | -ve | Detected | <40 | UD | <55 |
| A30 | 36 | TDF+3TC+EFV | N/A | T1 | 0 | -ve | No sample | | No sample | |
|  |  |  | N/A | T2 | 1.8 | -ve | Detected | 41 | UD | <110 |

^a^Lower limit of quantification and lower limit of detection varied based on the dilution factor; where the HIV-1 RNA was qualitatively detected the estimated levels are indicated. Abbreviations: ART=antiretroviral therapy; TDF=tenofovir disoproxil fumarate; 3TC=lamivudine; EFV=efavirenz; UD=undetected. Schistosomiasis status: +ve SEM = positive semen microscopy; +ve UF = positive urine filtration; +ve PCR = positive real-time polymerase chain reaction; -ve = negative for all the three tests, namely UF, SEM, and PCR.

**Supplementary Table 3.** Characteristics of the fourteen patients with concordant HIV-1 RNA detection in both seminal fluid and plasma.

| **ID** | **Age (years)** | **ART regimen** | **ART duration (years)** | **Timepoint** | **Follow-up time (months)** | **Schistosomiasis status** | **HIV-1 RNA** | | | |
| --- | --- | --- | --- | --- | --- | --- | --- | --- | --- | --- |
|  |  |  |  |  |  |  | **Plasma** | **Copies/ml** | **Seminal fluid** | **Copies/ml^a^** |
| A01 | 46 | TDF+3TC+EFV | 1.7 | T1 | 0 | +ve PCR | UD | <22 | UD | <110 |
|  |  |  | 1.8 | T2 | 13.0 | -ve | UD | <22 | UD | <55 |
| A06 | 47 | TDF+3TC+EFV | 11.8 | T1 | 0 | + SEM | UD | <22 | UD | <55 |
|  |  |  | 12.0 | T2 | 1.9 | -ve | UD | <22 | UD | <55 |
|  |  |  | 12.1 | T3 | 3.7 | -ve | UD | <22 | UD | <55 |
|  |  |  | 12.4 | T4 | 6.7 | +ve SEM | UD | <22 | UD | <55 |
|  |  |  | 12.9 | T5 | 12.9 | -ve | UD | <22 | UD | <55 |
| A10 | 25 | TDF+3TC+EFV | N/A | T1 | 0 | +ve PCR | UD | <22 | UD | <55 |
| A18 | 42 | TDF+3TC+EFV | 1.5 | T1 | 0 | -ve | UD | <22 | UD | <55 |
|  |  |  | 1.6 | T2 | 1.8 | -ve | UD | <22 | UD | <55 |
|  |  |  | 2.6 | T3 | 11.2 | -ve | No sample | | No sample | |
| A19 | 43 | TDF+3TC+EFV | 4.9 | T1 | 0 | -ve | No sample | | No sample | |
|  |  |  | 5.1 | T2 | 12.5 | -ve | UD | <22 | UD | <55 |
| A21 | 23 | TDF+3TC+EFV | 0.1 | T1 | 0 | -ve | Quantified | 26900 | Quantified | 4840 |
| A22 | 25 | TDF+3TC+EFV | 5.4 | T1 | 0 | -ve | UD | <22 | UD | <55 |
|  |  |  | 5.5 | T2 | 0.9 | -ve | UD | <22 | UD | <55 |
|  |  |  | 5.6 | T3 | 2.5 | -ve | UD | <22 | UD | <55 |
|  |  |  | 5.9 | T4 | 5.4 | -ve | UD | <22 | UD | <55 |
| A24 | 22 | TDF+3TC+EFV | N/A | T1 | 0 | -ve | UD | <22 | UD | <55 |
| A25 | 57 | TDF+3TC+EFV | 11.4 | T1 | 0 | -ve | No sample | | No sample | |
|  |  |  | 11.6 | T2 | 1.8 | -ve | UD | <22 | UD | <55 |
|  |  |  | 12.0 | T3 | 6.7 | -ve | UD | <22 | UD | <55 |
| A26 | 61 | TDF+3TC+EFV | 5.5 | T1 | 0 | -ve | UD | <22 | UD | <55 |
| A27 | 52 | TDF+3TC+NVP | 8.5 | T1 | 0 | -ve | UD | <22 | UD | <55 |
| A28 | 50 | TDF+3TC+EFV | 11.4 | T1 | 0 | -ve | No sample | | No sample | |
|  |  |  | 11.6 | T2 | 1.8 | -ve | UD | <22 | UD | <55 |
|  |  |  | 11.8 | T3 | 3.7 | -ve | UD | <22 | UD | <55 |
| A29 | 49 | TDF+3TC+EFV | 11.2 | T1 | 0 | -ve | UD | <22 | UD | <55 |
| A31 | 52 | TDF+3TC+EFV | 12.2 | T1 | 0 | -ve | No sample | | No sample | |
|  |  |  | 12.3 | T2 | 1.8 | -ve | UD | <22 | UD | <220 |
|  |  |  | 12.5 | T3 | 3.6 | -ve | UD | <22 | UD | <110 |
|  |  |  | 13.2 | T4 | 13.1 | -ve | UD | <22 | UD | <110 |

References

1. Villa G, Abdullahi A, Owusu D, Smith C, Azumah M, Sayeed L, et al. Determining virological suppression and resuppression by point-of-care viral load testing in a HIV care setting in sub-Saharan Africa. EClinicalMedicine. 2020;18:100231.

2. Cepheid. Xpert® HIV-1 Viral Load United States of America2018 [22/11/2019]. Available from: <https://www.cepheid.com/en/cepheid-solutions/clinical-ivd-tests/virology/xpert-hiv-1-viral-load>.

3. Xpert HIV-1 QUAL datasheet. [Internet]. 2018. Available from: <https://p.cdn.net/sde8xc/Cepheid-Xpert_hiv_1_qual_Brochure_CE-IVD-3053_English>.

4. WHO. WHO Prequalification of in Vitro Diagnostics. Public report product: Xpert HIV Qual assay WHO reference number: PQDx 0259-070-00. World Health Organization, Geneva, Switzerland. 2017.
